# Self-relevance and the activation of attentional networks

**DOI:** 10.1177/17470218221112238

**Published:** 2022-07-24

**Authors:** Saga Svensson, Marius Golubickis, Sam Johnson, Johanna K Falbén, C Neil Macrae

**Affiliations:** 1 School of Psychology, University of Aberdeen, Aberdeen, UK; 2 Department of Psychology, University of Warwick, Coventry, UK

**Keywords:** Self-prioritisation, Attention Network Test, executive control, conflict resolution, spotlight

## Abstract

Recent theoretical accounts maintain that core components of attentional functioning are preferentially tuned to self-relevant information. Evidence in support of this viewpoint is equivocal, however, with research overly reliant on personally significant (i.e., familiar) stimulus inputs (e.g., faces, forenames) and a diverse range of methodologies. Addressing these limitations, here we utilised arbitrary items (i.e., geometric shapes) and administered the Attention Network Test (ANT) to establish the extent to which self-relevance (vs friend-relevance) moderates the three subsystems of attentional functioning—alerting, orienting, and executive control. The results revealed that only executive control was sensitive to the meaning of the stimuli, such that conflict resolution was enhanced following the presentation of self-associated compared with friend-associated shapes (i.e., cues). Probing the origin of this effect, a further computational analysis (i.e., Shrinking Spotlight Diffusion Model analysis) indicated that self-relevance facilitated the narrowing of visual attention. These findings highlight when and how the personal significance of otherwise trivial material modulates attentional processing.

A prominent assertion in social-cognitive research is that, compared with items associated with other people (e.g., friend, mother, stranger), personally meaningful stimuli are prioritised during attentional processing (Conway & Pleydell-Pearce, 2000; Humphreys & Sui, 2016; Oyserman et al., 2012; Sui & Humphreys, 2015; Sui & Rotshtein, 2019). As Sui and Rotshtein (2019) have argued, “Human attention is tuned by self-related information” (p. 148). Given the pivotal status that self-relevant stimuli (e.g., one’s partner, purse, pizza) occupy in daily life, this privileged processing is to be expected (Constable et al., 2014, 2019; Schäfer et al., 2015, 2016). What is somewhat surprising therefore is that, despite extensive empirical efforts, the exact manner in which self-relevance impacts attention remains poorly understood. Two factors have contributed to this situation. First, a troublesome stimulus confound has called into question the alleged potency of self-related items in perception/attention (but see Sui et al., 2012; Woźniak & Knoblich, 2019). Second, inadequate theoretical consideration has been given to different aspects of attention and how they may (or indeed may not) be modulated by material associated with the self (but see Sui & Rotshtein, 2019). Responding to these limitations, here we explored the effects of self-relevance in a single task context—absent problematic stimuli—using a methodology capable of probing core components of attention. Our overarching objective was to clarify when and how self-relevance affects attentional processing (see also Orellana-Corrales et al., 2020, 2021).

According to Posner and Petersen’s (1990) influential account, attention comprises three functionally and anatomically distinct networks that support the operations of alerting, orienting, and executive control (Corbetta & Shulman, 2002; Petersen & Posner, 2012; Posner & Rothbart, 2007; Posner et al., 2016). Working automatically, the alerting network moderates arousal and vigilance, enabling attention to be sustained over periods of time. In contrast, through the voluntary direction of attention to specific locations, modalities, or objects of interest, the orienting network facilitates the prioritisation of sensory inputs. Finally, the executive control network supports goal preservation and the top-down regulation of task-related interference and error. Supported by different regions of the brain and engaging divergent neurochemical systems, these attentional networks underpin the maintenance of a state of vigilance/alertness, the enhancement of stimulus processing, and the resolution of conflict (Petersen & Posner, 2012; Posner & Rothbart, 2007).

Given the flexibility of self-function and the pivotal status of attentional processing in this regard (Humphreys & Sui, 2016; Sui & Humphreys, 2015), the extent to which self-relevance influences the various components of attention is of considerable theoretical significance. Indeed, writing on this topic, Sui and Rotshtein (2019) recently advanced an interesting observation. Based on an inspection of the available evidence, they concluded that self-relevance acts as a global modulator of stimulus processing, affecting the operation of all three attentional networks. That is, self-relevance enhances alerting, orienting, and executive control. Crucially, however, although the extant literature appears to support this viewpoint, it does so with an important caveat. As virtually all research to date has investigated aspects of attentional functioning using personally meaningful stimulus materials—notably faces or forenames (e.g., self-face vs friend-face)—it leaves open the possibility that the reported effects were driven by the familiarity rather than the self-relevance of the items (e.g., Alexopoulos et al., 2012; Brédart et al., 2006; Devue & Brédart, 2008; Liu et al., 2016; Moray, 1959; Sui et al., 2006; Sun et al., 2016; Tacikowski & Nowicka, 2010; Tong & Nakayama, 1999; Wojcik et al., 2018; but see Golubickis & Macrae, 2021; Macrae et al., 2017, 2018; Sui et al., 2009). Furthermore, as alerting, orienting, and executive control have been studied using an assortment of paradigms and dependent measures (Sui & Rotshtein, 2019), it remains unclear which aspects of attention were activated during the respective tasks. To provide a precise account of how self-relevance influences attentional functioning, what is needed is a single task in which the tripartite components of attention can be assessed simultaneously, with the attentional networks activated by stimuli absent pre-existing self-associations. Usefully, the Attention Network Test (ANT) offers just such an opportunity (Fan et al., 2002, 2005).

Developed by Fan et al. (2002), through the amalgamation of spatial cueing and flanker methodologies (B. A. Eriksen & Eriksen, 1974; Posner, 1980), the ANT provides a behavioural measure of the efficiency of the three attentional networks within a single task. In standard versions of the paradigm, participants are required to identify a central target that is flanked by compatible (e.g., > > > > >) or incompatible (e.g., < < > < <) distractors (B. A. Eriksen & Eriksen, 1974), with stimulus arrays appearing either above or below fixation. Additional cueing conditions are included to activate the alerting and orienting networks, with the executive control network triggered by target-flanker incompatibility (see Figure 1). In the no-cue condition, information signalling when and where the stimuli will appear is absent, thereby creating task-related uncertainty. In the alerting-cue conditions, in contrast, either a centre or double cue is presented. These cues indicate when the stimuli will appear but give no information about the spatial location of the items. Finally, in the orienting-cue condition, a single spatial cue is presented that reveals both when and where the stimuli will appear. Activation of the attentional networks is established by comparing the response times (RTs) observed in the ANT across the different cueing and stimulus conditions (i.e., alerting network = RT_no cue_ − RT_double cue_, orienting network = RT_centre cue_ − RT_spatial cue_, executive control network = RT_incompatible_ − RT_compatible_). Adopting this methodology or closely related variants, the dynamics of attentional functioning has been elucidated across a range of domains and populations (Arora et al., 2020; Posner et al., 2016).

**Figure 1.**
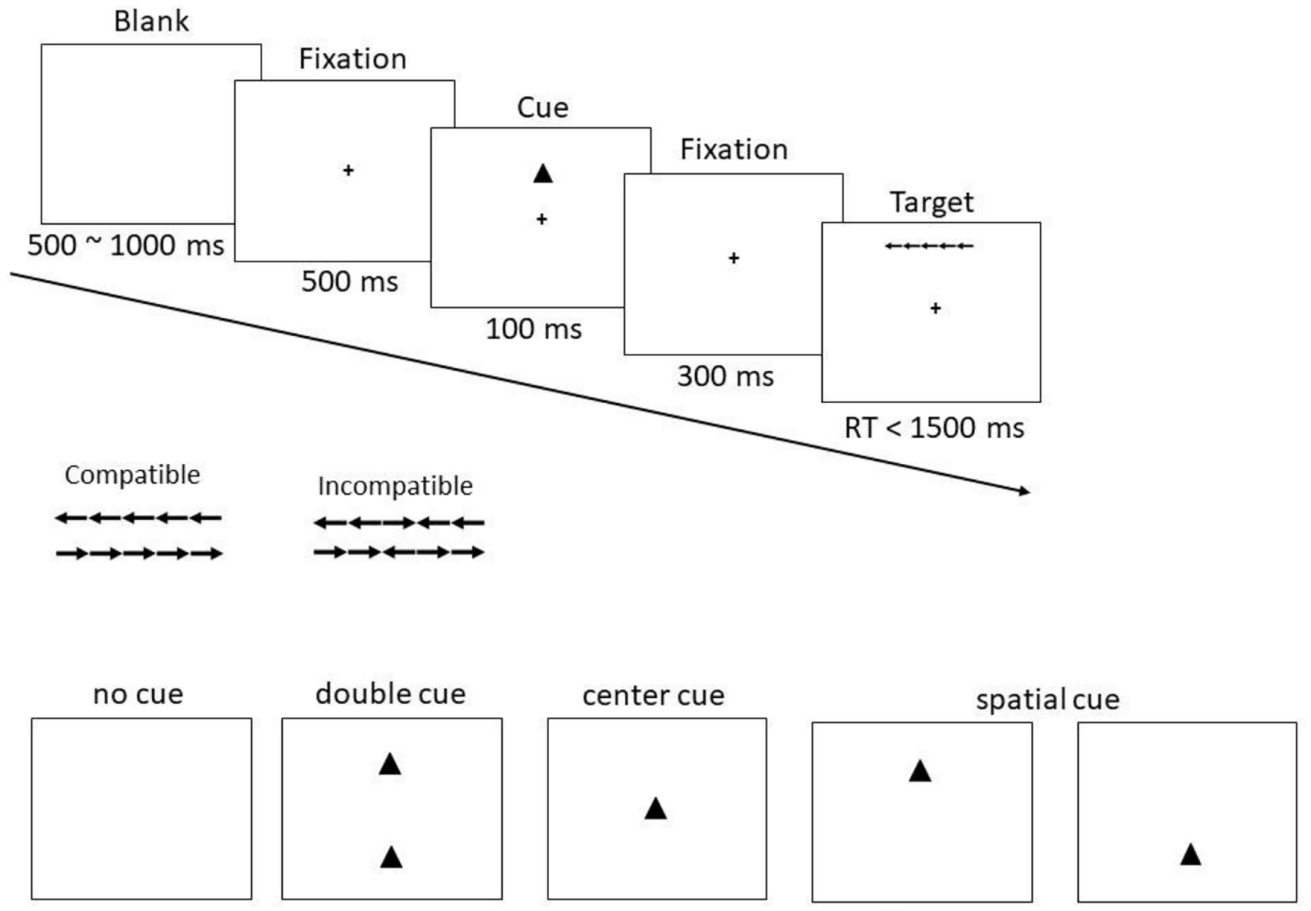
Schematic example of the procedure and timings for the Attention Network Test.

Using a modified version of the ANT, here we explored the extent to which self-relevance activates the attentional networks that support alerting, orienting, and executive control when the target stimuli have no pre-existing association with the self. Importantly, based on previous work investigating self-prioritisation, prior to performing the ANT participants learned target-shape associations (e.g., self = triangle, friend = square), pairings that were subsequently probed in a shape-label matching task (Sui et al., 2012). This task was undertaken to establish the existence of a self-prioritisation effect (SPE) prior to the geometric shapes serving as cues in the ANT. Thus, using arbitrary stimulus materials in a single task setting, the current experiment tested the hypothesis that self-relevance enhances performance in all three attentional networks (Sui & Rotshtein, 2019).

## Method

### Participants and design

Seventy participants were recruited (47 female, *M*_age_ = 22.63, *SD* = 3.18) using the Prolific platform for online testing (www.prolific.co), with each receiving compensation at the rate of £7.50/h.^1^ Informed consent was obtained from participants prior to the commencement of the experiment and the protocol was reviewed and approved by the Ethics Committee at the School of Psychology, University of Aberdeen, UK. The experiment had a 2 (Shape Association: self or friend) × 4 (Cue: centre or double or spatial or none) × 2 (Flanker: compatible or incompatible) repeated-measures design. Based on related research (Golubickis & Macrae, 2021), to establish if self-relevance moderated task performance (i.e., 2 × 2 repeated-measures interaction), a sample of 70 participants afforded 89% power for the detection of a medium effect size (i.e., *d* = .50; PANGEA, v .0.2).

### Stimulus materials and procedure

On accessing the experiment online, participants were told the study comprised two tasks, a shape-association task and an arrow-identification task. Prior to the shape-association task, participants were informed the computer would randomly assign a geometric shape (i.e., square or triangle) to denote them, and another shape to represent their best friend. They then pressed spacebar on the keyboard and the screen displayed which shapes designated self and best friend, respectively (e.g., you = square, friend = triangle). Further instructions explained that they would be presented with a shape (i.e., square or triangle) and a label (i.e., self or friend) and their task was simply to indicate, via a button press as quickly and accurately as possible, whether the shape and label matched or mismatched the previously learned associations (Sui et al., 2012). Responses were given using two keys on the keyboard (i.e., V & B). Key-response mappings were counterbalanced across participants and the labels “matching” and “nonmatching” were located on the screen, on the same side as the associated buttons on the keyboard, to serve as reminders throughout the task.

In the shape-association task, each trial began with a central fixation cross displayed for 500 ms, after which it disappeared and was replaced by a shape and label appearing above and below the fixation cross, respectively. The shape and label remained on the screen for 100 ms, after which the screen turned blank for 1,100 ms or until a response was made. Feedback was provided after each trial, lasting for 500 ms. The screen remained blank for a variable period of 500–1,000 ms before the next trial commenced. The stimuli consisted of a black square and triangle (i.e., 150 × 150 pixels, presented at 5% of each participant’s respective screen size) that were displayed on a white background. Participants initially performed 12 practice trials followed by a block of 120 experimental trials. Half of the trials comprised matching shape-label pairs, and half nonmatching pairings. The order of the trials was randomised. On completion of the task, participants were given further instructions regarding the second activity they were to perform.

The second task comprised a modified ANT (Fan et al., 2002). Participants were instructed they would see a row of five arrows and their task was to indicate, via button press, in which direction the central target arrow was pointing (i.e., leftwards or rightwards) while maintaining fixation on the central cross on the screen (see Figure 1). The flankers consisted of four arrows (two to the left and two to the right) that pointed either in the same direction as the target arrow (i.e., compatible trial) or in the opposite direction (i.e., incompatible trial). Responses were given using two keys on the keyboard (i.e., C & M) and key-response mappings were displayed on the screen throughout the task. Each target presentation was preceded by one of four cue conditions. In the no-cue condition, the fixation cross remained on the screen and the target stimulus appeared either above or below fixation. In the centre-cue condition, a cue (i.e., square or triangle) appeared at fixation, followed by the target stimulus either above or below. In the double-cue condition, cues (2 squares or 2 triangles) appeared simultaneously at the target locations above and below fixation. Finally, in the spatial-cue condition, a cue (i.e., square or triangle) appeared at the location of the target stimulus. Cues were displayed for 100 ms, followed by the fixation cross for 300 ms, after which the target appeared and stayed on the screen until a response was made or 1,500 ms had elapsed. The inter-trial interval varied randomly between 500 and 1,000 ms. Participants completed 6 blocks of 64 trials, resulting in a total of 384 trials. On completion of the task, participants were thanked and debriefed.

## Results

### Shape-association task

Responses faster than 200 ms and timed out trials were excluded from the analysis, eliminating less than 1% of the overall data. Five participants (2 female) were excluded for failing to follow the instructions. A 2 (Shape Association: self or friend) × 2 (Matching Condition: matching or nonmatching) repeated-measures analysis of variance (ANOVA) was conducted on participants’ mean correct response times (RTs) and response accuracy (see Table 1). Analysis of the RTs yielded main effects of Shape Association, *F*(1, 64) = 30.85, *p* < .001, $\eta_{p}^{2}$ = .32, Matching Condition, *F*(1, 64) = 47.91, *p* < .001, $\eta_{p}^{2}$ = .43, and a significant Shape Association × Matching Condition interaction, *F*(1, 64) = 53.93, *p* < .001, $\eta_{p}^{2}$ = .46. Further analysis of the interaction revealed that, during matching trials, responses were faster to a self-associated compared with a friend-associated shape, *t*(64) = 7.59, *p* < .001, *d_z_* = .94, BF_10_ = 5.85 × 10^7^. No significant effects were observed on nonmatching trials.

**Table 1.** Mean response times (ms) and accuracy (%) as a function of Shape Association and Matching Condition.

	Shape Association	
Matching Condition	Self	Friend
Response time		
Matching	591 (109)	687 (122)
Nonmatching	708 (118)	698 (116)
Accuracy		
Matching	94 (8)	76 (15)
Nonmatching	85 (13)	83 (14)

Standard deviation in parentheses.

Analysis of response accuracy yielded a main effect of Shape Association, *F*(1, 64) = 90.95, *p* < .001, $\eta_{p}^{2}$ = .59, and a significant Shape Association × Matching Condition interaction, *F*(1, 64) = 32.41, *p* < .001, $\eta_{p}^{2}$ = .34. Further analysis of the interaction revealed that, during matching trials, accuracy was greater for responses towards a self-associated compared with a friend-associated shape, *t*(64) = 9.23, *p* < .001, *d_z_* = 1.15, BF_10_ = 20,170. No significant effects were observed on nonmatching trials.

Collectively, these findings confirm the emergence of a standard SPE during the shape-association task (Sui et al., 2012).

### Attention Network Test

Activation of each attentional network was calculated according to Fan et al. (2002), the analyses of which are summarised below (see Table 2).

**Table 2.** ANT performance (ms) and attentional network scores (ms).

	Shape Association			
	Self		Friend	
Flanker	Compatible	Incompatible	Compatible	Incompatible
Cue				
Centre	511 (68)	599 (79)	501 (68)	613 (101)
Double	502 (74)	596 (81)	496 (68)	598 (84)
Spatial	485 (68)	562 (76)	488 (68)	568 (83)
Attentional network				
Alerting	25 (33)		26 (30)	
Orienting	31 (30)		29 (32)	
Executive control	87 (33)		95 (37)	

ANT: Attention Network Test.

Standard deviation in parentheses.

#### Alerting

A one-way ANOVA comparing the double-cue to the no-cue condition revealed a significant difference in response time, confirming that the alerting network was activated during the task, *F*(1, 64) = 4.48, *p* = .036, $\eta_{p}^{2}$ = .034, BF_10_ = 1.41. To establish if alerting was moderated by Shape Association, a one-way ANOVA comparing the network scores for self-related and friend-related trials was undertaken. This yielded no significant difference, *F*(1, 64) = 0.06, *p* = .81, BF_01_ = 5.19, indicating that alerting was not modulated by self-relevance.

#### Orienting

A 2 (Shape Association: self or friend) × 2 (Cue: centre cue or spatial cue) revealed only a main effect of Cue, such that responses were faster following a spatial cue compared with a centre cue, *F*(1, 64) = 108.84, *p* < .001, $\eta_{p}^{2}$ = .63 (respective *M*s: 525 ms vs 554 ms). The failure to observe a significant Shape Association × Cue interaction showed that orienting was insensitive to the self-relevance of the shapes, *F*(1, 64) = 0.37, *p* = .54, BF_01_ = 20.97.

#### Executive control

A 2 (Shape Association: self or friend) × 2 (Flanker: compatible or incompatible) yielded a main effect of Flanker, *F*(1, 64) = 613.53, *p* < .001, $\eta_{p}^{2}$ = .61 (*M*s: compatible 498 ms vs incompatible 587 ms), and a significant Shape Association × Flanker interaction, *F*(1, 64) = 6.03, *p* = .017, $\eta_{p}^{2}$ = .09. To further investigate the interaction, the respective network scores for trials following self- and friend-associated shapes were calculated and compared. This revealed that conflict was significantly lower when target stimuli were preceded by a self-related compared with a friend-related shape, *t*(64) = 2.46, *p* = .008, *d_z_* = .30, BF_10_ = 4.32 (respective *M*s: 87 ms vs 95 ms).

Thus, while activation of the alerting, orienting, and executive control networks was observed in the current experiment, only executive control was sensitive to the self-relevance of the cues.

### Additional analysis

To probe whether the benefits of self-relevance on shape-label matching and executive control represent distinct or related effects, an additional correlational analysis was undertaken. This yielded no significant correlation between the measures, *r*(64) = –.13, *p* = .31, BF_01_ = 3.88.

### Shrinking spotlight diffusion model analysis

To elucidate how self-relevance influenced executive control, data (RT & accuracy) were submitted to an additional Shrinking Spotlight (SSP) Diffusion Model analysis (White & Curl, 2018; White et al., 2011). An extension of the Drift Diffusion Model (DDM) of decision-making, the SSP was developed to identify the latent cognitive processes that underpin performance during flanker tasks. The model assumes that information is continually sampled from a target until sufficient evidence has been gathered to select a response (i.e., reach one of the decision thresholds). A primary strength of the model is that it is able to account for changes in both response time and accuracy simultaneously and it has been applied successfully in previous work exploring the effects of self-relevance on attentional breadth (Golubickis & Macrae, 2021). Departing from the standard DDM, a basic assumption of the SSP is that the accumulation of decisional evidence (i.e., drift rate) varies over time as a function of how attention is allocated during the flanker task. In other words, the resolution (i.e., breadth) of the attentional spotlight moderates task performance. At the early stages of processing attention is diffuse, such that flankers contribute significantly to the drift rate. As the task unfolds, through contraction of the spotlight, attention focuses more narrowly on the target, thereby reducing flanker interference. Crucially, the SSP captures this rate of attentional shrinkage (White et al., 2011).

The SSP parameters associated with the latent cognitive operations underpinning task performance include boundary separation (*a*), perceptual strength (*p*), non-decision time (*Ter*), spotlight width (*sd_a_*), and shrinking rate (*r_d_*). Boundary separation (*a*) estimates the distance between the two decision thresholds and thus indicates how much evidence is required before a response is selected (i.e., response caution). Perceptual strength (*p*) reflects the efficiency of visual processing (i.e., the contribution each stimulus makes towards faster decision-making), such that large (vs small) values signal more rapid information uptake. The duration of all non-decisional processes is given by the *Ter* parameter, which indicates differences in stimulus encoding and response execution. Finally, the spotlight width (*sd_a_*) and shrinking rate (*r_d_*) parameters collectively index attentional control during the flanker task. At the beginning of a trial, the *sd_a_* estimates the initial distribution of attention, and *r_d_* represents the speed at which the spotlight contracts on the central target. Together, these parameters probe the extent to which attentional control is enhanced by a focused spotlight and/or rapid shrinking rate (White et al., 2011).

**Figure 2.**
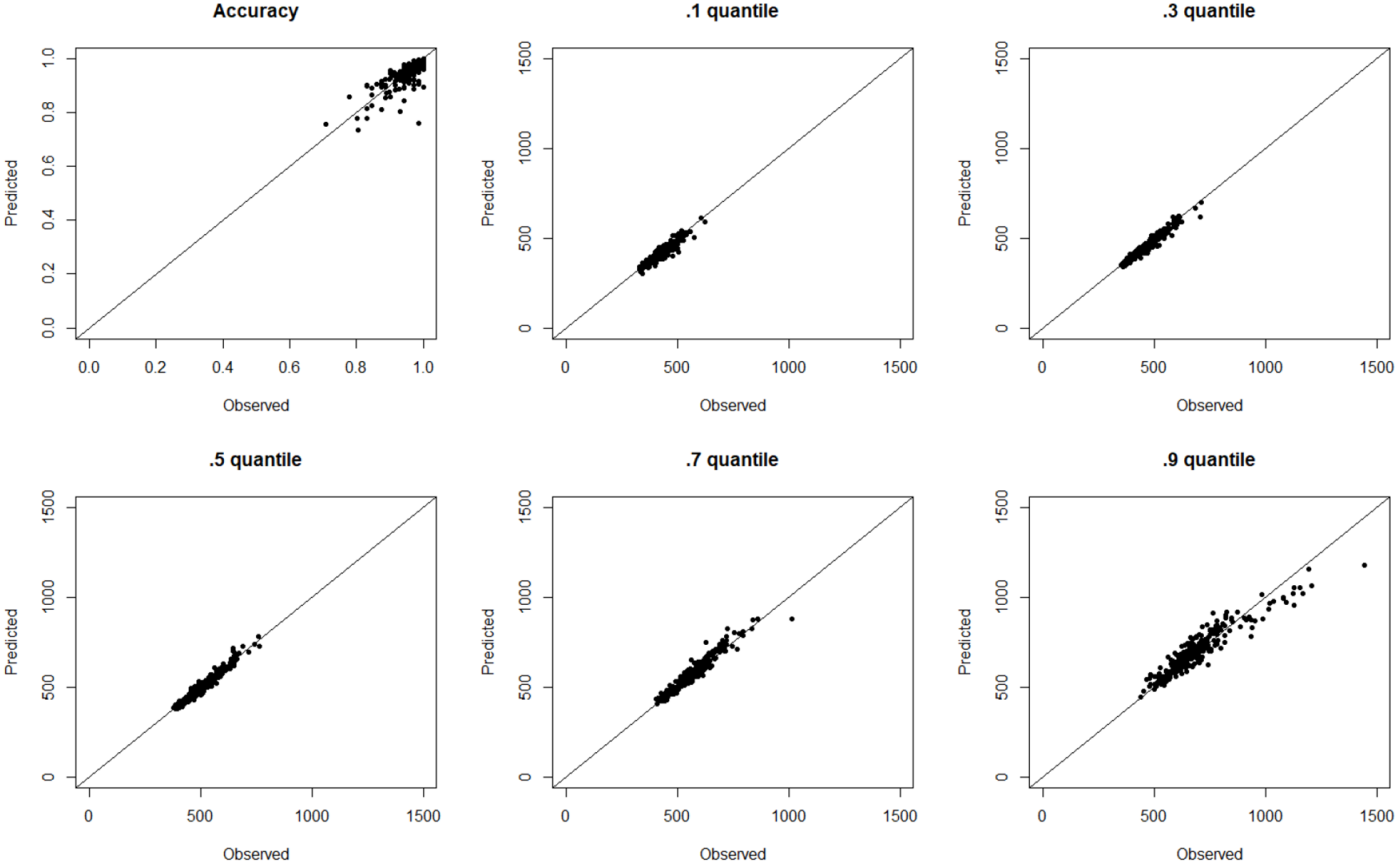
Fit quality from the SSP analysis. Observed responses are plotted against predicted responses from the best fitting SSP parameters for accuracy and the RT quantiles (ms).

To estimate the parameters of the SSP, data (i.e., RT quantiles and accuracy) were submitted to the fitting procedure adopted by Golubickis and Macrae (2021). With the exception of the spotlight width (*sd_a_*), all parameters (*a, p, Ter, s_d_*) varied as a function of Shape Association (i.e., self vs friend) and were fitted separately for each participant. The spotlight width (*sd_a_*) was fixed at a value of 1 (Servant & Evans, 2020). Thus, the SSP parameters for each participant and Shape Association reflected the best fitting estimates for both compatible and incompatible trials simultaneously (White et al., 2011). The quality of model fit was evaluated by simulating data sets from the estimated parameters and then comparing these with the observed data (i.e., posterior predictive check). With nearly complete overlap between the simulated estimates and observed values, this demonstrated good model fit (see Figure 2).

**Figure 3.**
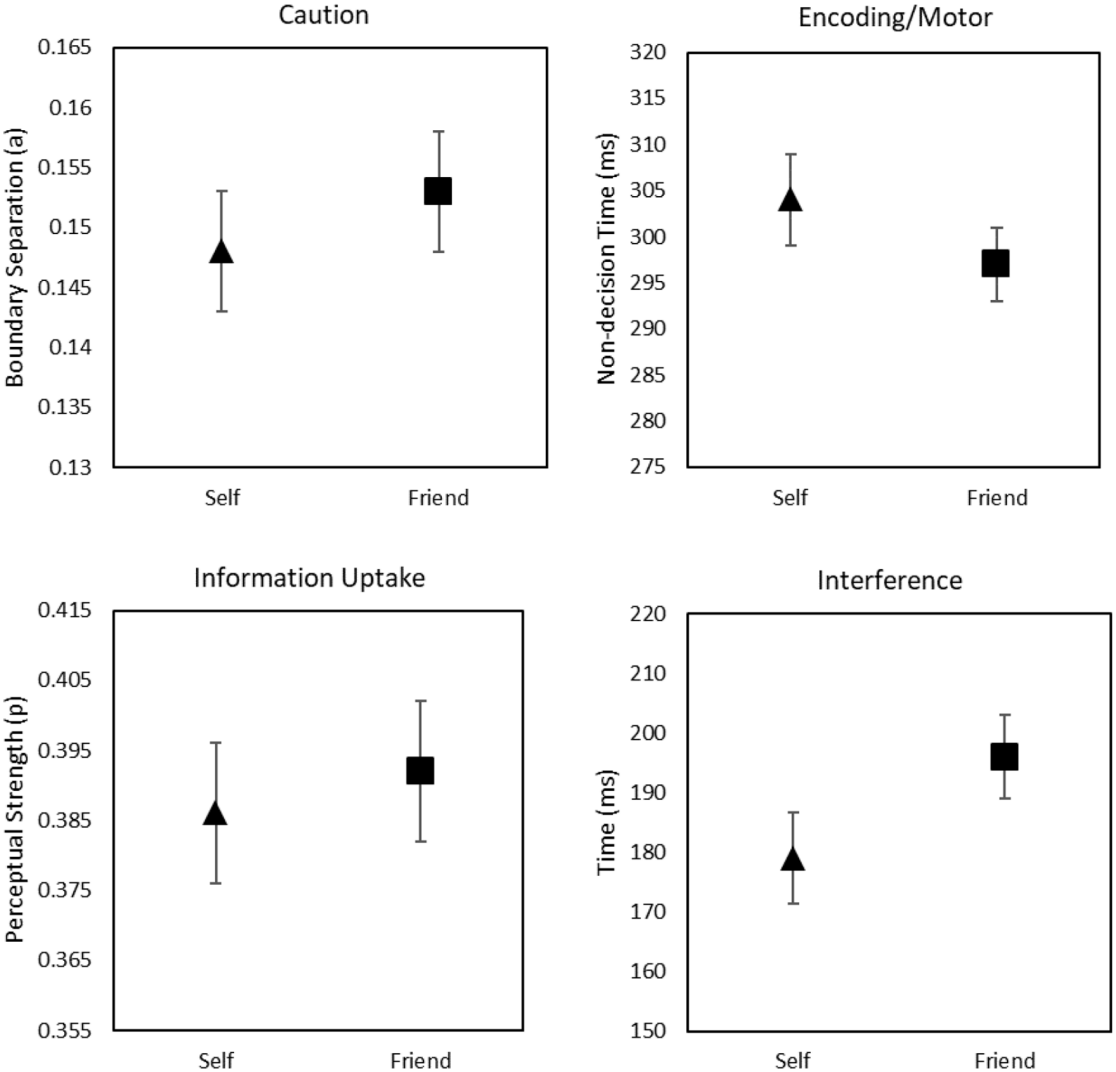
Shrinking spotlight parameters as a function of Shape Association. Error bars represent ±1 SEM.

The SSP parameter estimates were submitted to a paired-sample (Shape Association: self vs friend) *t*-test (two-tailed). The analysis yielded no significant effects on estimates of boundary separation, *a, t*(64) = –0.83, *p* = .41, *d_z_* = .10, BF_01_ = 5.29, non-decision time, *Ter, t*(64) = 1.34, *p* = .19, *d_z_* = .16, BF_01_ = 3.15, or perceptual strength, *p, t*(64) = 0.60, *p* = .55, *d_z_* = .07, BF_01_ = 6.18. The efficiency of attentional control was evaluated by calculating the ratio between the spotlight width and shrinking rate parameters (i.e., *sd_a_/r_d_*). The resulting measure captures the interference time, specifically the time needed to focus attention fully on the target in the stimulus array, with smaller (vs larger) values indicating a better ability to engage selective attention and reduce flanker interference (White et al., 2011). The analysis of this parameter revealed that less time was needed to focus attention (i.e., shrink the spotlight) on the target following self-relevant (*M* = 179 ms, *SD* = 62 ms) compared with friend-relevant (*M* = 195 ms, *SD* = 56 ms) shapes, *t*(64) = 2.46, *p* = .02, *d_z_* = .31, BF_10_ = 2.21. This confirms that self-relevance facilitated attentional control (see Figure 3).

## Discussion

Using a modified ANT, here we explored the extent to which self-relevance influences core facets of attentional functioning: specifically, alerting, orienting, and executive control (Corbetta & Shulman, 2002; Petersen & Posner, 2012; Posner & Rothbart, 2007; Posner et al., 2016). Notwithstanding the contention that self-relevance enhances the operation of all three attentional subsystems (Sui & Rotshtein, 2019), only executive control yielded a significant effect. During the ANT, attentional control was facilitated when flanker arrays were preceded by self-associated compared with friend-associated shapes. Probing the origin of this effect, an additional computational analysis (i.e., SSP diffusion model—Golubickis & Macrae, 2021; White & Curl, 2018; White et al., 2011) revealed that self-relevance affected the performance by speeding the narrowing of attention on the to-be-judged target (i.e., shrinkage of the attentional spotlight), thereby reducing flanker interference (C. W. Eriksen & St. James, 1986). In contrast, neither alerting nor orienting was sensitive to the meaning of the shapes (Cohen et al., 2011; Orellana-Corrales et al., 2021). In addition, and interestingly, the benefits of self-relevance on shape-label matching (i.e., self-prioritisation) and executive control were not correlated, indicating the independence of these variants of self-bias (Amodeo et al., 2021; Nijhof et al., 2020).

Before considering the implications of these findings, an important point must be made. Based on the current results, we are not suggesting that personally consequential material is incapable of modulating activity in all three attentional networks. Indeed, it would be surprising—and somewhat suboptimal—if attention operated in this way. To navigate the challenges of daily living, people are unquestionably finely tuned to personally relevant stimuli, be they parents, pets, or possessions. Moreover, depending on the task context and processing goals in place, these items likely facilitate multiple aspects of attentional functioning. Crucially, however, such effects can be attributed to the familiarity of the stimuli (e.g., own mother vs friend’s mother—Sui et al., 2012; Woźniak & Knoblich, 2019) rather than their self-relevance per se. In this respect, what is noteworthy about recent theoretical accounts of self-function is the assertion that, because of the potency of self-relevance, attentional benefits emerge even when the items in question are entirely arbitrary (e.g., geometric shapes, colours) and have no pre-existing association with the self (Humphreys & Sui, 2016; Sui & Humphreys, 2015, 2017). As measured by the ANT (Fan et al., 2002), however, the current findings failed to support this viewpoint at least for alerting and orienting, thus undermining the contention that inconsequential self-associated stimuli exert an obligatory influence on the activation of all three attentional networks (Sui & Humphreys, 2015; Sui & Rotshtein, 2019).^2^ Instead, the results resonate with the observation that self-relevance exerts greatest influence on decisional and response-related operations, rather than the earlier stages of attentional processing (e.g., Caughey et al., 2021; Constable et al., 2019; Golubickis et al., 2018; Siebold et al., 2015; Stein et al., 2016; Wade & Vickery, 2018).

The demonstration that self-relevance only influenced the efficiency of executive control raises several interesting issues. In closely related research, Golubickis et al. (2021) considered a basic facet of executive function—response inhibition (Diamond, 2013; Friedman & Miyake, 2004). Using a stop-signal task (Verbruggen & Logan, 2008), the work explored the ease with which responses to self-relevant (vs friend-relevant) objects could intentionally be stopped. Highlighting the benefits of personal relevance, performance was facilitated when participants were required to withhold responses to self-associated compared with friend-associated stimuli. In other words, self-relevance enhanced the attentional operations that underpin response suppression (Petersen & Posner, 2012; Posner & Petersen, 1990; Posner et al., 2016). Exploring another core aspect of executive control—the resolution of response conflict—the current results similarly demonstrated the benefits of self-relevance (i.e., reduced flanker interference). On this occasion, however, self-associated (vs friend-associated) cues facilitated performance in an ANT in which the flanker arrays (i.e., arrows) held no meaning for participants. Thus, extending previous research on executive control, self-relevance enhanced the attentional processing of arbitrary stimuli (cf. Golubickis & Macrae, 2021).

Central to the generation of the current effects is an executive-control system that is geared to optimising self-directed behaviour through the reduction of interference (i.e., conflict) from potentially competing thoughts, memories, and actions (Hofmann et al., 2012). Take action control, for example. Given the demonstration that visuomotor processing is enhanced when interacting with self-relevant compared with other-relevant objects (Constable et al., 2011, 2014), it is unsurprising that response inhibition operates in a similar way (Golubickis et al., 2021). Of course, what is noteworthy about the current findings is that executive control was not directed towards self-relevant stimuli, rather personal relevance triggered the enhanced attentional processing—in the form of reduced flanker interference—of subsequently presented material. What this suggests is that self-relevance has the capacity to increase attentional gain, hence processing efficiency, for yet-to-be encountered stimuli. A commonly reported finding is that attentional processing is enhanced for expected (i.e., predicted) stimulus inputs (Bar, 2007; Summerfield & Egner, 2009, 2016). During the ANT, it is possible that self-relevant (vs friend-relevant) cues elicited (i.e., primed) an expectancy that related items would follow. Although this was not in fact the case, the cue-associated attentional gain that was triggered nevertheless carried over to the subsequent flanker task, facilitating conflict resolution through shrinkage of the attentional spotlight (Golubickis & Macrae, 2021; White & Curl, 2018; White et al., 2011). A useful task for future research will therefore be to explore exactly when and for how long self-relevant cues influence the processing of personally irrelevant stimuli in this way.

Additional consideration should also be given to the effects that self-relevance exerts on other executive operations. Broadly speaking, executive control refers to a raft of higher order cognitive abilities that enable people to pursue their goals in a flexible manner (Miller & Cohen, 2001; Norman & Shallice, 1986). In addition to response inhibition, the updating/monitoring of working memory representations and mental set/task shifting are other prominent executive processes (Miyake et al., 2000). Exploring the dynamics of self-function, recent research has demonstrated the automatic prioritisation of self-associated representations in working memory, an effect that is causally underpinned by activity in regions of the prefrontal cortex (Yin et al., 2019, 2021). Based on these findings, Yin et al. (2021) have argued that the ventromedial prefrontal cortex (vmPFC) biases working memory towards self-associated items, which in turn enhances the modulation of attentional operations to maintain these representations in memory (Humphreys & Sui, 2016; Sui & Humphreys, 2015). In so doing, the strength of self-associated material in working memory ultimately facilitates executive control (Hofmann et al., 2012). Extending this general line of inquiry, it would be interesting to explore the extent to which the self-relevance of stimuli influences the efficiency of task (or mental set) switching when processing objectives vary in respect to their pertinence to the self or working memory resources are constrained (Caughey et al., 2021; Dalmaso et al., 2019; Falbén et al., 2019; Woźniak & Knoblich, in press). Work of this kind would further elucidate how self-relevance influences core facets of executive control.

Focusing on the ANT, future research should also examine whether self-relevance drives potential interactions among the three attentional subsystems. A limitation of the standard ANT (Fan et al., 2002) is that because the same cue is used to measure alerting and orienting, it is not possible to establish if the associated networks interact in a meaningful way. In addition, as the spatial cue is always predictive with respect to the location of the target, the task does not allow assessment of the reorientation of attention following the presentation of invalid cues. Rectifying these issues, Callejas et al. (2004) developed a new version of the paradigm—the Attention Network Test for Interactions (ANT-I)—in which the double cue was replaced with an alerting tone and the spatial cue was predictive of the target location on only 50% of the trials. Critically, this modified task structure enables the three networks and their interactions to independently be assessed (Callejas et al., 2004, 2005; Fuentes & Campoy, 2008). Adopting such a methodology, additional work could extend the current inquiry by probing how (and with what effects) the attentional networks are coordinated during self-referential processing (Humphreys & Sui, 2016; Sui & Humphreys, 2015; Sui & Rotshtein, 2019).

Using the ANT in combination with arbitrary stimulus materials (i.e., geometric shapes), here we explored the extent to which self-relevance (vs friend-relevance) moderated activation of the three subsystems of attentional functioning—alerting, orienting, and executive control (Corbetta & Shulman, 2002; Petersen & Posner, 2012; Posner & Rothbart, 2007; Posner et al., 2016). The results revealed that only executive control was sensitive to the personal significance of the stimuli, such that conflict resolution was enhanced following the presentation of self-associated compared with friend-associated shapes (i.e., cues). Examining the origin of this effect, a bespoke computational analysis (i.e., SSP diffusion model analysis, White & Curl, 2018) indicated that self-relevant (vs friend-relevant) cues facilitated the narrowing of visual attention. Collectively, these findings highlight when and how the personal significance of otherwise meaningless stimuli modulates attentional processing.

## Notes

1 The data generated during the current experiment are available at the Open Science Framework at the following link: https://osf.io/zyauc/.

2 The objective of the current inquiry was to explore, in a single task (i.e., ANT), the extent to which self-relevance affects the activation of three attentional subsystems. Notwithstanding the reported results (i.e., only executive control was sensitive to self-relevance), it remains to be seen whether arbitrary self-associated items would moderate alerting and orienting in the specific tasks that have been designed to explore these operations. To date, the evidence is mixed. Whereas Sui et al. (2009) failed to observe a reflexive orienting effect when arrows were associated with the self (vs friend) and Orellana-Corrales et al. (2020, 2021) found no effect of newly self-associated (vs other-associated) stimuli on attentional capture using a dot-probe task, Macrae et al. (2018) demonstrated that, through their effects on transient attention, self-relevant (vs other-relevant) cues enhanced contrast sensitivity. This topic awaits further empirical investigation and clarification.

## References

[bibr1-17470218221112238] Alexopoulos T. Mueller D. Ric F. Marendaz C. (2012). I, me, mine: Automatic attentional capture by self-related stimuli. European Journal of Social Psychology, 42, 770–779.

[bibr2-17470218221112238] Amodeo L. Wiersema J. R. Brass M. Nijhof A. D. (2021). A comparison of self-bias measures across cognitive domains. BMC Psychology, 9, Article 132. 10.1186/s40359-021-00639-x PMC8414869 34479639

[bibr3-17470218221112238] Arora S. Lawrence M. A. Klein R. M. (2020). The attention network test database: ADHD and cross-cultural applications. Frontiers in Psychology, 11, Article 388. 10.3389/fpsyg.2020.00388 PMC7119191 32292363

[bibr4-17470218221112238] Bar M. (2007). The proactive brain: Using analogies and associations to generate predictions. Trends in Cognitive Science, 11, 280–289. 10.1016/j.tics.2007.05.005 17548232

[bibr5-17470218221112238] Brédart S. Delchambre M. Laureys S. (2006). One’s own face is hard to ignore. Quarterly Journal of Experimental Psychology, 59, 46–52. 10.1080/17470210500343678 16556557

[bibr6-17470218221112238] Callejas A. Lupiáñez J. Fuenes M. J. Tudela P. (2005). Modulations among the alerting, orienting, and executive control networks. Experimental Brain Research, 167, 27–37. 16021429 10.1007/s00221-005-2365-z

[bibr7-17470218221112238] Callejas A. Lupiáñez J. Tudela P. (2004). The three attentional networks: On their independence and interactions. Brain and Cognition, 54, 225–227. 15050779 10.1016/j.bandc.2004.02.012

[bibr8-17470218221112238] Caughey S. Falbén J. K. Tsamadi D. Persson L. M. Golubickis M. Macrae C. N. (2021). Self-prioritization during stimulus processing is not obligatory. Psychological Research, 85, 503–508. 31919569 10.1007/s00426-019-01283-2 PMC7900024

[bibr9-17470218221112238] Cohen N. Henik A. Mor N. (2011). Can emotion modulate attention? Evidence for reciprocal links in the attentional network test. Experimental Psychology, 58, 171–179. 20705545 10.1027/1618-3169/a000083

[bibr10-17470218221112238] Constable M. D. Kritikos A. Bayliss P. (2011). Grasping the concept of personal property. Cognition, 119, 430–437. 21377147 10.1016/j.cognition.2011.02.007

[bibr11-17470218221112238] Constable M. D. Kritikos A. Lipp O. V. Bayliss P. (2014). Object ownership and action: The influence of social context and choice on the physical manipulation of personal property. Experimental Brain Research, 232, 3749–3761. 25138911 10.1007/s00221-014-4063-1

[bibr12-17470218221112238] Constable M. D. Welsh T. N. Huffman G. Pratt J. (2019). I before U: Temporal order judgements reveal bias for self-owned objects. Quarterly Journal of Experimental Psychology, 72, 589–598. 10.1177/1747021818762010 29431023

[bibr13-17470218221112238] Conway M. A. Pleydell-Pearce C. W. (2000). The construction of autobiographical memories in the self-memory system. Psychological Review, 107, 261–288. 10789197 10.1037/0033-295x.107.2.261

[bibr14-17470218221112238] Corbetta M. Shulman G. L. (2002). Control of goal-directed and stimulus-driven attention in the brain. Nature Reviews, 3, 201–215. 10.1038/nrn755 11994752

[bibr15-17470218221112238] Dalmaso M. Castelli L. Galfano G. (2019). Self-related shapes can hold the eyes. Quarterly Journal of Experimental Psychology, 72, 2249–2260. 10.1177/1747021819839668 30852940

[bibr16-17470218221112238] Devue C. Brédart S. (2008). Attention to self-referential stimuli: Can I ignore my own face? Acta Psychologica, 128, 290–297. 18413272 10.1016/j.actpsy.2008.02.004

[bibr17-17470218221112238] Diamond A. (2013). Executive functions. Annual Review of Psychology, 64, 135–168. 10.1146/annurev-psych-113011-143750 PMC4084861 23020641

[bibr18-17470218221112238] Eriksen B. A. Eriksen C. W. (1974). Effects of noise letters upon the identification of a target letter in a non-search task. Perception & Psychophysics, 16, 143–149.

[bibr19-17470218221112238] Eriksen C. W. St. James J. D. (1986). Visual attention within and around the field of focal attention: A zoom lens model. Perception & Psychophysics, 40, 225–240. 3786090 10.3758/bf03211502

[bibr20-17470218221112238] Falbén J. K. Golubickis M. Balseryte R. Persson L. M. Tsamadi D. Caughey S. Macrae C. N. (2019). How prioritized is self-prioritization during stimulus processing? Visual Cognition, 27, 46–51.

[bibr21-17470218221112238] Fan J. McCandliss B. D. Fossella J. Flombaum J. Posner M. I. (2005). The activation of attentional networks. NeuroImage, 26, 471–479. 15907304 10.1016/j.neuroimage.2005.02.004

[bibr22-17470218221112238] Fan J. McCandliss B. D. Sommer T. Raz A. Posner M. I. (2002). Testing the efficiency and independency of attentional networks. Journal of Cognitive Neuroscience, 14, 340–347. 11970796 10.1162/089892902317361886

[bibr23-17470218221112238] Friedman N. P. Miyake A. (2004). The relations among inhibition and interference control functions: A latent-variable analysis. Journal of Experimental Psychology: General, 133, 101–135. 14979754 10.1037/0096-3445.133.1.101

[bibr24-17470218221112238] Fuentes L. J. Campoy G. (2008). The time course of alerting effect of over orienting in the network test. Experimental Brain Research, 185, 667–672. 17989966 10.1007/s00221-007-1193-8

[bibr25-17470218221112238] Golubickis M. Falbén J. K. Cunningham W. A. Macrae C. N. (2018). Exploring the self-ownership effect: Separating stimulus and response biases. Journal of Experimental Psychology: Learning, Memory and Cognition, 44, 295–306. 28933899 10.1037/xlm0000455

[bibr26-17470218221112238] Golubickis M. Macrae C. N. (2021). That’s me in the spotlight: Self-relevance modulates attentional breadth. Psychonomic Bulletin and Review, 28, 1915–1922. 34159529 10.3758/s13423-021-01964-3

[bibr27-17470218221112238] Golubickis M. Persson L. M. Falbén J. K. Macrae C. N. (2021). On stopping yourself: Self-relevance facilitates response inhibition. Attention, Perception, & Psychophysics, 4, 1416–1423. 10.3758/s13414-021-02248-7 33665767

[bibr28-17470218221112238] Hofmann W. Schmeichel B. J. Baddeley A. D. (2012). Executive functions and self-regulation. Trends in Cognitive Sciences, 16, 174–180. 22336729 10.1016/j.tics.2012.01.006

[bibr29-17470218221112238] Humphreys G. W. Sui J. (2016). Attentional control and the self: The self-attention network (SAN). Cognitive Neuroscience, 7, 5–17. 25945926 10.1080/17588928.2015.1044427

[bibr30-17470218221112238] Liu M. He X. Rotshtein P. Sui J. (2016). Dynamically orienting your own face facilitates the automatic attraction of attention. Cognitive Neuroscience, 7, 37–44. 25978648 10.1080/17588928.2015.1044428 PMC4873716

[bibr31-17470218221112238] Macrae C. N. Visokomogilski A. Golubickis M. Cunningham W. Sahraie A. (2017). Self-relevance prioritizes access to visual awareness. Journal of Experimental Psychology: Human Perception and Performance, 43, 438–443. 28240929 10.1037/xhp0000361

[bibr32-17470218221112238] Macrae C. N. Visokomogilski A. Golubickis M. Sahraie A. (2018). Self-relevance enhances the benefits of attention on perception. Visual Cognition, 26, 475–481.

[bibr33-17470218221112238] Miller E. K. Cohen J. D. (2001). An integrative theory of prefrontal cortex function. Annual Review of Neuroscience, 24, 167–202. 10.1146/annurev.neuro.24.1.167 11283309

[bibr34-17470218221112238] Miyake A. Friedman N. P. Emerson M. J. Witzki A. H. Howerter A. Wager T. D. (2000). The unity and diversity of executive functions and their contributions to complex “frontal lobe” tasks: A latent variable analysis. Cognitive Psychology, 41, 49–100. 10945922 10.1006/cogp.1999.0734

[bibr35-17470218221112238] Moray N. (1959). Attention in dischotic listening: Affective cues and the influence of instructions. Quarterly Journal of Experimental Psychology, 11, 56–60.

[bibr36-17470218221112238] Nijhof A. D. Shapiro K. L. Catmur C. Bird G. (2020). No evidence for a common self-bias across cognitive domains. Cognition, 197, Article 104186. 10.1016/j.cognition.2020.104186 31954993

[bibr37-17470218221112238] Norman D. A. Shallice T. (1986). Attention to action: Willed and automatic control of behaviour. In Davidson R. J. Schwartz G. E. Shapiro D. (Eds.), Consciousness and self-regulation (pp. 1–14). Plenum Press.

[bibr38-17470218221112238] Orellana-Corrales G. Matschke C. Wesslein A. (2020). Does self-associating a geometric shape immediately cause attentional prioritization? Experimental Psychology, 67, 335–348. 33661037 10.1027/1618-3169/a000502

[bibr39-17470218221112238] Orellana-Corrales G. Matschke C. Wesslein A. (2021). The impact of newly self-associated pictorial and letter-based stimuli in attention holding. Attention, Perception, & Psychophysics, 83, 2729–2743. 10.3758/s13414-021-02367-1 34426930

[bibr40-17470218221112238] Oyserman D. Elmore K. Smith G. (2012). Self, self-concept, and identity. In Leary M. R. Tangney J. P. (Eds.), Handbook of self and identity (2nd ed., pp. 69–104). Guildford Press.

[bibr41-17470218221112238] Petersen S. E. Posner M. I. (2012). The attention system of the human brain: 20 years after. Annual Review of Neuroscience, 35, 73–89. 10.1146/annurev-neuro-062111-150525 PMC3413263 22524787

[bibr42-17470218221112238] Posner M. I. (1980). Orienting of attention. Quarterly Journal of Experimental Psychology, 32A, 3–25. 10.1080/00335558008248231 7367577

[bibr43-17470218221112238] Posner M. I. Petersen S. E. (1990). The attention system of the human brain. Annual Review of Neuroscience, 13, 25–42. 10.1146/annurev.ne.13.030190.000325 2183676

[bibr44-17470218221112238] Posner M. I. Rothbart M. K. (2007). Research on attention networks as a model for the integration of psychological science. Annual Review of Clinical Psychology, 58, 1–23. 10.1146/annurev.psych.58.110405.085516 17029565

[bibr45-17470218221112238] Posner M. I. Rothbart M. K. Voelker P. (2016). Developing brain networks of attention. Current Opinions in Pediatrics, 28, 720–724. 10.1097/MOP.0000000000000413 PMC5257020 27552068

[bibr46-17470218221112238] Servant M. Evans N. J. (2020). A diffusion model analysis of the effects of aging in the Flanker Task. Psychology and Aging, 35(6), 831–849. 10.1037/pag0000546 32658539

[bibr47-17470218221112238] Schäfer S. Wentura D. Frings C. (2015). Self-prioritization beyond perception. Experimental Psychology, 62, 415–425. 27120563 10.1027/1618-3169/a000307

[bibr48-17470218221112238] Schäfer S. Wesslein A. Spence C. Wentura D. Frings C. (2016). Self-prioritization in vision, audition, and touch. Experimental Brain Research, 234, 2141–2150. 26979440 10.1007/s00221-016-4616-6

[bibr49-17470218221112238] Siebold A. Weaver M. D. Donk M. van Zoest W. (2015). Social salience does not transfer to oculomotor visual search. Visual Cognition, 23, 989–1019.

[bibr50-17470218221112238] Stein T. Siebold A. van Zoest W. (2016). Testing the idea of privileged awareness of self-relevant information. Journal of Experimental Psychology: Human Perception and Performance, 42, 303–307. 26727020 10.1037/xhp0000197

[bibr51-17470218221112238] Sui J. He X. Humphreys G. W. (2012). Perceptual effects of social salience: Evidence from self-prioritization effects on perceptual matching. Journal of Experimental Psychology: Human Perception and Performance, 38, 1105–1117. 22963229 10.1037/a0029792

[bibr52-17470218221112238] Sui J. Humphreys G. W. (2015). The integrative self: How self-reference integrates perception and memory. Trends in Cognitive Sciences, 19, 719–728. 26447060 10.1016/j.tics.2015.08.015

[bibr53-17470218221112238] Sui J. Liu C. H. Wang L. Han S. (2009). Attention orientation induced by temporarily established self-referential cues. Quarterly Journal of Experimental Psychology, 62, 844–849. 10.1080/17470210802559393 19132633

[bibr54-17470218221112238] Sui J. Rotshtein P. (2019). Self-prioritization and the attentional systems. Current Opinion in Psychology, 29, 148–152. 30913475 10.1016/j.copsyc.2019.02.010

[bibr55-17470218221112238] Sui J. Zhu Y. Han S. (2006). Self-face recognition in attended and unattended conditions: An event-related brain potential study. NeuroReport, 17, 423–427. 16514370 10.1097/01.wnr.0000203357.65190.61

[bibr56-17470218221112238] Summerfield C. Egner T. (2009). Expectation (and attention) in visual cognition. Trends in Cognitive Sciences, 13, 403–409. 19716752 10.1016/j.tics.2009.06.003

[bibr57-17470218221112238] Summerfield C. Egner T. (2016). Feature-based attention and feature-based expectation. Trends in Cognitive Sciences, 20, 401–404. 27079632 10.1016/j.tics.2016.03.008 PMC4875850

[bibr58-17470218221112238] Sun Y. Fuentes L. J. Humphreys G. W. Sui J. (2016). Try to see it my way: Domain-specific embodiment enhances self and friend-biases in perceptual matching. Cognition, 153, 108–117. 27183397 10.1016/j.cognition.2016.04.015 PMC6020994

[bibr59-17470218221112238] Tacikowski P. Nowicka A. (2010). Allocation of attention to self-name and self-face: An ERP study. Biological Psychology, 84, 318–324. 20298741 10.1016/j.biopsycho.2010.03.009

[bibr60-17470218221112238] Tong F. Nakayama K. (1999). Robust representation for faces: Evidence from visual search. Journal of Experimental Psychology: Human Perception and Performance, 25, 1016–1035. 10464943 10.1037//0096-1523.25.4.1016

[bibr61-17470218221112238] Verbruggen F. Logan G. D. (2008). Automatic and controlled response inhibition: Associative learning in the go/no-go and stop-signal paradigms. Journal of Experimental Psychology: General, 137, 649–672. 18999358 10.1037/a0013170 PMC2597400

[bibr62-17470218221112238] Wade G. L. Vickery T. J. (2018). Target self-relevance speeds up visual search responses but does not improve search efficiency. Visual Cognition, 26, 563–582.

[bibr63-17470218221112238] White C. N. Curl R. (2018). Cueing effects in the attentional network test: A spotlight diffusion model analysis. Computational Brain & Behavior, 1, 59–68.

[bibr64-17470218221112238] White C. N. Ratcliff R. Starns J. J. (2011). Diffusion models of the flanker task: Discrete versus gradual attentional selection. Cognitive Psychology, 63, 210–238. 21964663 10.1016/j.cogpsych.2011.08.001 PMC3195995

[bibr65-17470218221112238] Wojcik M. J. Nowicka M. N. Kotlewska I. Nowicka A. (2018). Self-face captures, holds, and biases attention. Frontiers in Psychology, 8, Article 2371. 10.3389/fpsyg.2017.02371 PMC5768651 29375456

[bibr66-17470218221112238] Woźniak M. Knoblich G. (2019). Self-prioritization of fully unfamiliar stimuli. Quarterly Journal of Experimental Psychology, 72, 2110–2120. 10.1177/1747021819832981 30727822

[bibr67-17470218221112238] Woźniak M. Knoblich G. (2022). Self-prioritization depends on assumed task-relevance of self-association. Psychological Research, 86, 1599–1614. 34491432 10.1007/s00426-021-01584-5

[bibr68-17470218221112238] Yin S. Bi T. Chen A. Egner T. (2021). Ventromedial prefrontal cortex drives the prioritization of self-associated stimuli in working memory. Journal of Neuroscience, 41, 2012–2023. 33462089 10.1523/JNEUROSCI.1783-20.2020 PMC7939096

[bibr69-17470218221112238] Yin S. Sui J. Chiu Y.-C. Chen A. Egner T. (2019). Automatic prioritization of self-referential stimuli in working memory. Psychological Science, 30, 415–423. 30653399 10.1177/0956797618818483

